# Early identification of LHON carriers may improve outcome

**Published:** 2019

**Authors:** Josef Finsterer

**Affiliations:** * Krankenanstalt Rudolfstiftung, Messerli Institute, Veterniary University of Vienna, Vienna, Austria

We read with interest about two brothers with Leber’s hereditary optic neuropathy (LHON) due to the ND1 variant m.3460G>A by Iorga et al. [**1**]. We have the following comments and concerns.

We do not agree with the notion that LHON is the most common mitochondrial disorder (MID) as indicated in the abstract. Much more prevalent than specific MIDs, including LHON, are non-specific mitochondrial multiorgan disorder syndromes (MIMODSs) [**2**]. MIMODSs are frequently missed and overlooked for years, since the clinical presentation does not fit to any of the known specific mitochondrial syndromes of which about 50 have been clearly delineated so far. A further reason for overlooking MIMODSs is the broad intra- and inter-familial phenotypic heterogeneity, why heredity of the condition is frequently not immediately recognised.

A further shortcoming of the study by Iorga et al. is that they do not mention if the variant m.3460G>A occurred in a heteroplasmic or homoplasmic distribution. In the majority of the cases, primary LHON mutations occur in the homoplasmic form but rare exceptions have been described [**3**]. It would be also interesting to know if the variant was also detected in tissues other than lymphocytes, such as urinary epithelial cells, fibroblasts, muscle cells, or hair follicles and if heteroplasmy rates differed from those in lymphocytes.

Another inadequacy of the study is that no family screening for the pathogenic variant had been carried out [**1**]. Accordingly, we do not know who else in the family carried the mutation. Since early initiation of idebenone may result in a better outcome of visual acuity than late initiation of treatment [**4**], it is crucial to recognise the condition at a pre-clinical or early clinical stage. Those carrying a primary LHON mutation need to be screened regularly not to miss the point at which carriers develop pre-clinical or clinical manifestations requiring immediate initiation of treatment. 

LHON may not only be mono-organ but in some cases also multi-organ with onset either already at the start of the ocular abnormalities or later during the disease course (LHON+). Organs/ tissues other than the retinal ganglion cells and the optic nerve affected in LHON are the central nervous system (psychomotor delay, dementia, epilepsy, leukoencephalopathy, posterior reversible encephalopathy syndrome (PRES), migraine, chorea, ataxia), the ears (hypoacusis), endocrine organs (diabetes, hypothyroidism, parathyroid dysfunction, pituitary adenoma), the heart (left ventricular hypertrabeculation/ noncompaction, dilated cardiomyopathy, supraventricular and ventricular arrhythmias, syncope, angina, sudden cardiac death), the bone-marrow (anemia), arteries (aortic stiffness), the kidneys (renal insufficiency), or the peripheral nerves (neuropathy) [**5**]. Were the two patients prospectively investigated for LHON+ and were other organs/ tissues affected?

Concerning the association between LHON and multiple sclerosis, as has been reported by Harding et al. [**6**], such an association is quite unlikely. Iorga et al. cite a study from 2000, which has not been confirmed. There is, however, a frequent secondary immune response to cell components affected by the metabolic breakdown. Organs in which a secondary immune response is obvious are the pancreas (aseptic, chronic pancreatitis), the submandibular glands (sialadenitis), the thyroid (Hashimoto or de Quervain thyroiditis), the colon (non-specific colitis), hepatocytes (immunehepatitis), synovial cells (synovitis, arthritis), or the glial cells (demyelination mimicking multiple sclerosis). Treatment of these secondary immune responses with immunosuppressants may exhibit a beneficial effect in some patients but in the majority of the cases, immunosuppressants are more harmful than beneficial. Which patients profit from such a treatment and who may develop side effects is difficult to predict.

In summary, the interesting report by Iorga et al. lacks information about the heteroplasmy rates of the variant, about the carriers’ status in relatives of the index patients, and prospective investigations for LHON+. Recognition of a LHON variant in the pre-clinical stage is essential to start idebenone as early as possible to improve the outcome. 

**Conflicts of interest**

There are no conflicts of interest.

**Funding**

No funding was received.

**Ethical approval**

The research has been given ethical approval. 
